# Nanotechnology-mediated podocyte injury repair: mechanistic exploration and therapeutic prospects

**DOI:** 10.1080/0886022X.2026.2673608

**Published:** 2026-05-24

**Authors:** Han Zhu, Pinyao Liang, Houjing Liu, Shulian Chen, Guobiao Liang

**Affiliations:** a Department of Urology, Affiliated Hospital of Zunyi Medical University, Zunyi, China; b The second Department of Urology, The First Affiliated Hospital of Kunming Medical University, Kunming, China; c School of Chemistry and Chemical Engineering, Guizhou University, Guiyang, China

**Keywords:** Nanotechnology, podocyte injury, kidney diseases, repair mechanisms

## Abstract

Podocyte injury serves as a central pathological driver in chronic kidney diseases (CKD), including diabetic kidney disease (DKD) and IgA nephropathy (IgAN). However, conventional therapies are still limited by poor targeting efficacy and systemic side effects. Nanotechnology provides transformative strategies *via* tunable nanomedicines, which enables precise podocyte repair through targeted drug delivery, antioxidant and anti-inflammatory effects, as well as gene regulation. This review exclusively focuses on the preclinical research progress in this field. Three categories of nanomaterials: peptide-drug conjugates (PDCs), polymeric nanoparticles (PNs), and inorganic nanomaterials (INs), exhibit distinct advantages: PDCs realize ligand-receptor-mediated precise targeting, PNs combine gene silencing with favorable biocompatibility, and INs rely on nanozyme activities to maintain redox homeostasis. Despite remarkable preclinical advances, challenges remain in optimizing targeting efficiency, overcoming biological barriers, and ensuring the long-term biosafety of nanomedicines. Future directions will center on stimuli-responsive nanosystems, multimodal therapies, organ-on-a-chip models, and sustainable manufacturing, which are critical for bridging the gap between preclinical research and clinical translation. This review highlights the great potential of nanotechnology in revolutionizing podocyte-targeted therapy and provides a comprehensive preclinical basis for addressing unmet clinical needs in CKD management *via* interdisciplinary innovation.

## Graphical Abstract

**Figure:**
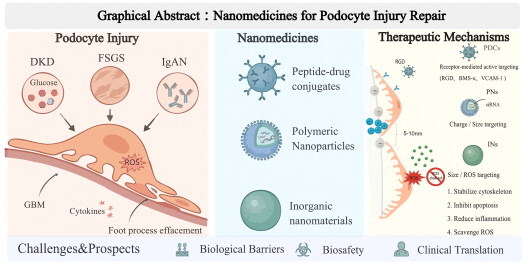

## 1. Introduction

The kidneys maintain systemic metabolic homeostasis through precise regulation of glomerular filtration, tubular reabsorption, and renal circadian rhythms [1]. However, this sophisticated regulatory system faces significant challenges: The incidence of acute kidney injury (AKI) continues to rise among hospitalized patients, particularly in critically ill populations, with mortality rates increasing in parallel with AKI stage severity [2,3]. Notably, chronic kidney disease (CKD) now affects over 850 million individuals globally and is projected to become the fifth leading cause of death worldwide by 2050 [4–6]. These epidemiological trends underscore the limitations of conventional therapeutic approaches in addressing the complex challenges posed by modern renal pathologies.

Podocytes, highly specialized cells within the glomerulus, serve as a ‘structural-functional dual hub’, playing a pivotal role in maintaining glomerular filtration barrier (GFB) integrity and normal renal physiological activities. Podocyte depletion and structural–functional abnormalities are closely associated with renal injury [7–10]. Podocytes consist of a cell body, primary processes, and foot processes. Primary processes extend from the cell body and further branch into interdigitating foot processes, which adhere to the glomerular basement membrane (GBM) [11–13]. The interaction between podocytes and GBM is critical for GFB function, involving intricate mechanisms such as cell-matrix adhesion, signaling pathway regulation, and dynamic mechanical support [14–17]. Adjacent foot processes are interconnected by slit diaphragms (∼25 nm in width), whose integrity relies on the dynamic equilibrium of the nephrin-CD2AP-podocin protein complex [18,19]. Podocyte injury and loss represent a central pathological process in various kidney diseases, driven by genetic, immunological, infectious, metabolic, and mechanical factors [20].

Current therapeutic strategies for podocyte injury include renin-angiotensin-aldosterone system (RAAS) inhibitors, SGLT2 inhibitors, and calcineurin inhibitors [21–28]. Emerging targeted agents, such as APOL1 inhibitors, complement inhibitors, and mitochondria-targeted drugs, are also under investigation [29–31]. However, both conventional and novel therapies face limitations, including limited efficacy, drug resistance, poor targeting precision, and significant adverse effects. A major challenge lies in achieving precise drug delivery to podocytes, which resides on the outer aspect of the GBM. Nanomedicines, leveraging their unique physicochemical properties—such as nanoscale size effects, high surface-to-volume ratios, and surface modifiability—offer innovative solutions to restore podocyte homeostasis. Given the promising application prospects of nanotechnology in podocyte injury repair and the current lack of a systematic summary of its research progress, mechanistic exploration, and translational challenges, this review aims to comprehensively elucidate the advances in nanotechnology-mediated podocyte injury repair, clarify the design features and therapeutic outcomes of different types of nanomaterials for podocyte protection, dissect the core molecular mechanisms and key regulatory pathways through which nanomedicines repair podocyte injury, and systematically analyze the current bottlenecks in this field—from targeting efficiency and biosafety to clinical translation.

## 2. Podocyte injury

### 2.1. Diabetic kidney disease

DKD is a leading cause of CKD and end-stage renal disease (ESRD) [32]. In diabetes, dysfunction of the GFB directly contributes to proteinuria, with podocyte injury—as a core component of the GFB—serving as a pivotal mechanism driving DKD progression. Current evidence indicates that podocyte injury in DKD involves multifaceted pathological factors, including metabolic dysregulation, hemodynamic stress, oxidative injury, and genetic susceptibility [33].

Glucotoxicity and lipotoxicity synergistically induce podocyte apoptosis, detachment, and dysfunction. Under chronic hyperglycemia, aberrant glucose metabolism activates the polyol and hexosamine pathways, leading to increased reactive oxygen species (ROS) production and elevated oxidative stress levels, not only directly damage podocyte structure and function but also exacerbate local inflammation through pro-inflammatory cytokine release [34,35]. Lipotoxicity disrupts the glycoprotein network, causing foot process effacement. This process is accompanied by enhanced caspase-3 activation, mitochondrial dysfunction, and ROS accumulation, ultimately triggering podocyte apoptosis, Furthermore, lipotoxicity impairs insulin signaling, reducing podocyte insulin sensitivity and perpetuating a vicious cycle of metabolic derangement and pathological injury [36–39], while Ang II-mediated RAAS activation and ET-1 upregulation induce glomerular hypertension and nephrin/podocin downregulation, driving foot process remodeling [33].

### 2.2. Podocytopathies

Podocytopathies are a cluster of kidney diseases characterized by primary podocyte injury, mainly including Minimal Change Disease (MCD) and Focal Segmental Glomerulosclerosis (FSGS). MCD is featured by extensive foot process effacement with mild podocyte density reduction [40–42]; FSGS is divided into primary, genetic, and secondary subtypes, with pathological progression from podocyte injury to severe depletion [43], and its pathogenic mechanisms include four core aspects: circulating permeability factor-mediated immune dysregulation (with soluble urokinase-type plasminogen activator receptor, suPAR, being one of the most well-studied circulating factors that directly injures podocytes and disrupts GFB function) [44,45], genetic mutations of GFB-related genes (NPHS1, APOL1, etc.), toxic/infectious injury, and compensatory hyperfiltration [46–51]. Podocyte density and total podocyte number per glomerulus are important prognostic indicators [52], and circulating anti-nephrin antibodies are potential biomarkers for podocytopathy monitoring [53].

### 2.3. IgA nephropathy

IgA Nephropathy (IgAN), the most prevalent primary glomerulonephritis worldwide, leads to ESRD in 20%–40% of patients within 20 years [54], with definitive diagnosis relying on renal biopsy and mesangial IgA deposition as the hallmark pathological feature. Its classic ‘multi-hit’ pathogenesis is based on aberrant Gd-IgA1-mediated immune dysregulation [55]: Gd-IgA1 elevation (Hit 1) forms immune complexes with autoantibodies in genetically susceptible individuals (Hit 2), and the deposition of these complexes in the mesangium (Hit 3) activates mesangial cells and the complement cascade, releasing pro-inflammatory/fibrotic factors and ultimately causing secondary podocyte injury and GFB disruption.

## 3. Nanomedicines for podocyte repair

Nanomedicine refers to therapeutic agents engineered through nanotechnology into nanoscale particles or integrated with carrier materials to form drug delivery systems. Its core advantages lie in leveraging the unique physicochemical properties of nanomaterials to enhance drug delivery efficiency, biocompatibility, and targeting precision [56,57]. Since the first synthesis of colloidal gold in 1951 and the introduction of liposome technology in 1965, nanomedicine has evolved rapidly. In 1995, Doxil—the first liposomal nanomedicine—received FDA approval for ovarian cancer treatment, marking its clinical translation [58–60]. Nanomedicine preparation methods are broadly classified into chemical synthesis, physical synthesis, and biosynthesis [61,62]. In CKD therapeutic research, chemical synthesis dominates due to its high controllability and reproducibility.

### 3.1. Types and targeting strategies

**Figure 1.**
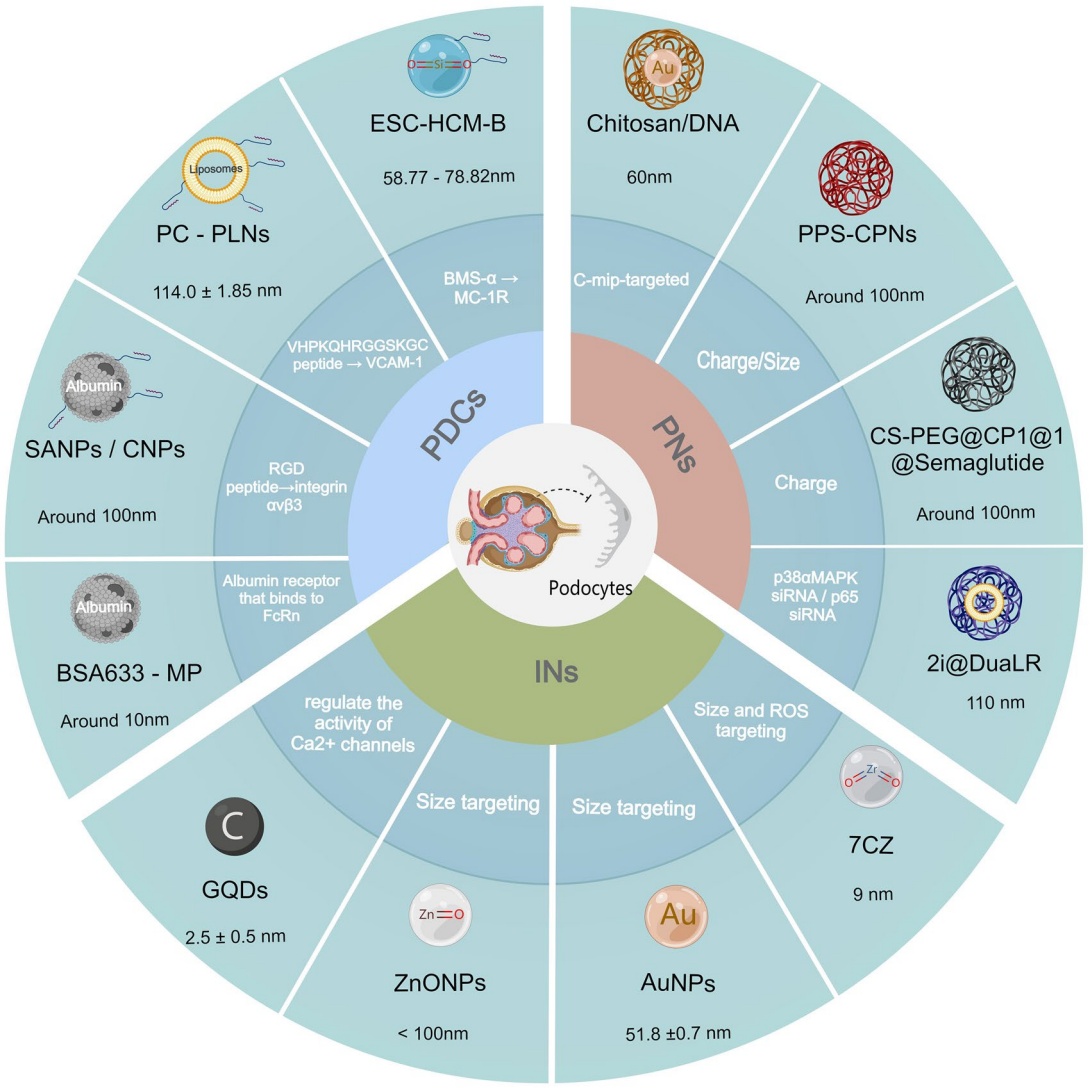
Schematic illustration of the core composition, particle size, and targeting strategies of three types of nanomaterials for podocyte injury repair (peptide-drug conjugates, PDCs; polymeric nanoparticles, PNs; inorganic nanomaterials, INs). This schematic is constructed based on the experimental data and findings from the referenced studies in this review, illustrating the characteristic particle sizes, key targeting ligands/receptors, and major targeting approaches of representative nanomaterials for podocyte protection.

In light of the distinct podocyte injury characteristics in different types of renal diseases, differential strategies are required for the design and targeting of nanomedicines. As shown in Figure 1, peptide-drug conjugates (PDCs) and polymeric nanoparticles (PNs) can achieve targeted intervention against specific molecular targets *via* ligand modification, gene delivery, and other approaches, while inorganic nanomaterials (INs) serve as universal intervention agents for the repair of podocyte injury of all types by virtue of their intrinsic physicochemical properties. For DKD characterized by high integrin αvβ3 expression, RGD peptide-modified PDCs can be adopted to realize ligand-receptor mediated active targeting, which delivers tacrolimus to podocytes for cytoskeleton stabilization and mitochondrial damage inhibition [63]. For abnormal HDAC4/miRNA-30a expression, RGD-modified PNs can be constructed to enable precise siRNA/miRNA delivery and gene silencing, thereby suppressing high glucose-induced podocyte apoptosis [64]. For MCD/FSGS with abnormal MC-1R expression, mTORC1 pathway activation and immune injury mediated by circulating permeability factors, BMS-α ligand-modified PDCs can mediate the targeted delivery of glucocorticoids to inhibit the mTORC1 pathway and stabilize the podocyte cytoskeleton while reducing the systemic toxicity of glucocorticoids [65]; albumin receptor-targeted PDCs can deliver methylprednisolone to podocytes in a targeted manner *via* FcRn, and their small-size property can enhance glomerular enrichment efficiency [66]. For IgAN, in response to the high expression of VCAM-1, celastrol can be delivered *via* VCAM-1 ligand-modified PDCs to inhibit the NF-κB inflammatory pathway and reduce the release of inflammatory factors [67]. Alternatively, charge-size synergistically targeted PNs can be used for the delivery of siRNAs to silence inflammatory genes of p38αMAPK and p65; ROS-responsive PNs can also be constructed to inhibit complement activation locally in the mesangial area/podocytes, thus reducing immune complex deposition and extracellular matrix accumulation [68,69]. Unlike the disease-specific targeting of PDCs and PNs, inorganic nanomaterials have no specific target-binding capacity, but they can efficiently scavenge ROS through mechanisms such as nanozyme activity, and reverse podocyte cytoskeletal damage, mitochondrial dysfunction, and apoptotic processes mediated by oxidative stress. This effect can cover all pathological processes of podocyte injury accompanied by oxidative stress [70–73].

#### 3.1.1. PDCs: Precision targeting and efficient delivery

PDCs achieve targeted therapeutic delivery by chemically conjugating bioactive peptides to therapeutic agents, enabling selective binding to specific cell-surface receptors. For example, RGD peptide-modified tacrolimus-loaded nanoparticles (SANPs/CNPs, ∼100 nm) specifically target podocytes *via* integrin αvβ3-mediated endocytosis. These nanocarriers stabilize the podocyte cytoskeleton, mitigate mitochondrial damage, and suppress apoptosis in both *in vitro* and *in vivo* models [63].

Beyond RGD-based strategies, peptide-functionalized systems have been engineered to target VCAM-1 (e.g. VHPKQHRGGSKGC-modified celastrol-loaded PLNs, entrapment efficiency (EE) = 94.83 ± 0.87%, Drug Loading Capacity (DLC) = 2.83 ± 0.025%) and MC-1R (e.g. BMS-α-modified everolimus-loaded ESC-HCM-B, DLC = 16.245%), effectively inhibiting inflammation and apoptosis in LPS-induced podocytes and STZ-induced diabetic mice, respectively [67,74]. While PDCs offer notable advantages, including high targeting specificity and controllable drug release, their clinical translation is hindered by suboptimal peptide stability *in vivo*—strategies such as PEGylation have been explored to extend circulation half-life and improve pharmacokinetic profiles [67] (Table 1).

**Table 1.** PDCs Nanomaterials with podocyte protection effects.

Core composition	Size (nm)	Main targeting strategy	Drug loading	EE (%)	DLC (%)	Main mechanism of action	Verification model	Experimental phase	Refs.
(1) RGD-HSA-TAC SANPs (2) RGD-HSA-TAC CNPs	Around 100	RGD → αvβ3	Tacrolimus	/	SANPs:8.49 (2) CNPs:10.48	(1) Stabilize cytoskeleton (2) Alleviate mitochondrial damage (3) Suppression of Apoptosis	(1) Podocyte (HG+AGEs) (2) DM mouse model(STZ)	C + A₁	[63]
PC-PLNs	114.0 ± 1.85	VHPKQHRGGSKGC → VCAM-1	Celastrol	94.83 ± 0.87	2.83 ± 0.025	(1) Inhibition of inflammation (2) Suppression of apoptosis	Podocyte (LPS) (2) IgAN mouse model (3) CPN mouse model	C + A_2_	[67]
DTsiANp-cRGD/HDAC4	65.31 ± 1.83	RGD → αvβ3	HDAC4 siRNA	90 ± 2	/	Suppression of podocyte inflammation/apoptosis/autophagy by HDAC4 silencing	Podocyte (HG) (2) DM mouse model (STZ)	C + A₁	[64]
DTmiAnp-cRGD-30a mm	73.51 ± 1.43	RGD → αvβ3	miRNA-30a	/	/	Up-regulate the expression of miRNA-30a, thereby inhibiting the podocyte apoptosis	Podocyte (HG) (2) DM mouse model (STZ)	C + A₁	[75]
ESC-HCM-B	58.77–78.82	BMS-α → MC-1R	Everolimus	32.495	16.245	Stabilize cytoskeleton (2) Inhibits oxidative stress (3) mTORC1 inhibition	Podocyte (HG) (2) DM mouse model (STZ) (3) Lep−/− mice	C + A_2_	[74]
BSA633-MP	Around 10	Albumin receptor that binds to FcRn	Methylprednisolone	/	/	Reduce apoptosis of cells	Podocyte (PAN)	C	[66]
Dex/PFP@LIPs-BMS-α	190	BMS-α → MC-1R	Dexamethasone	84.95	/	Stabilize cytoskeleton (2) Suppression of apoptosis (3) Inhibits complement activation	Podocyte (PAN) (2) Rat model(PHN)	C + A₁	[65]

PDCs: Peptide-drug conjugates; EE: encapsulation efficiency; DLC: drug loading capacity; HG: high glucose; AGEs: advanced glycation end products; DM: diabetes mellitus; STZ: streptozotocin; IgAN: IgA nephropathy; CPN: crescentic glomerulonephritis; PAN: puromycin aminonucleoside; PHN: passive Heymann nephritis; Lep−: leptin knockout; C: *in vitro* only; A: *in vivo* only; C + A₁: *in vitro* + single animal model; C + A_2_: *in vitro* + two animal models.

#### 3.1.2. PNs: Multifunctional carriers and gene regulation

PNs utilize biocompatible polymers to encapsulate therapeutics *via* physical adsorption or covalent conjugation. Representative examples include 60 nm chitosan/DNA nanocomplexes delivering c-mip-targeting miRNA to alleviate podocyte inflammation and proteinuria [76]; 5–30 nm A4PEG5/20/CL10PEG20 nanoparticles (loaded with dexamethasone) stabilizing podocyte cytoskeletons *via* size targeting, with EE of 4.6%–9.1% and DLC of 0.6%–1.5% [77]; 100 nm PPS-CPNs/CLT (celastrol-loaded) achieving 91.86 ± 1.59% EE and 3.52 ± 0.17% DLC for anti-inflammatory/anti-apoptotic effects *via* charge/size targeting [69]; and 110 nm 2i@DuaLR (co-delivering p38α MAPK/p65 siRNA) with 93.75% EE suppressing inflammation and ECM deposition [68].

Collectively, PNs’ physicochemical tailorability enables targeted renal delivery, but balancing loading capacity and therapeutic efficacy remains critical for translating preclinical outcomes to clinical kidney disease therapies (Table 2).

**Table 2.** PNs Nanomaterials with podocyte protection effects.

Core composition	Size (nm)	Main targeting strategy	Drug loading	EE (%)	DLC (%)	Main mechanism of action	Verification model	Experimental phase	Ref.
(1) A4PEG5 (2) A4PEG20 (3) A4CL10PEG20	5–30	Size targeting	Dexamethasone	(1) A4PEG5:4.9 (2) A4PEG20:4.6 (3) A4CL10PEG20:9.1	(1) A4PEG5:0.8 (2) A4PEG20:0.6 (3) A4CL10PEG20:1.5	Stabilize the cytoskeleton	Podocyte (ADR) (2) Mouse model (ADR)	C + A₁	[77]
chitosan/DNA	60	c-mip-targeted chitosan-based RNAi nanocarrier	Plasmid DNA encoding miRNA	/	/	Inhibiting c-mip expression, stabilizing the cytoskeleton, and reducing proteinuria	(1) Podocyte (high expression of c-MIP) (2) Mouse model (LPS)	C + A₁	[76]
PPS-CPNs/CLT	Around 100	Charge/Size targeting	Celastrol	91.86 ± 1.59	3.52 ± 0.17	(1) Inhibition of inflammation (2) Suppression of apoptosis (3) Inhibits complement activation	(1) Podocyte cells (2) Rat model(PHN)	C + A₁	[69]
CS-PEG@CP1@1@Semaglutide	Around 100	Charge	Semaglutide	/	/	(1) Inhibition of inflammation (2) Inhibit podocyte apoptosis	(1) Podocyte (2) HK-2 cells	C	[78]
2i@DuaLR	110	Charge/Size targeting	p38αMAPK siRNA/p65 siRNA	93.75	/	(1) Inhibition of Inflammation (2) Reduction in ECM deposition (3) Decrease in immune complex deposition	(1) Podocyte (2) Rat model (PHN) (3) IgAN mouse model	C + A_2_	[68]
nCUR	270	The ligand gambogic acid (GA) enables active targeting to enhance its absorption across the intestinal barrier.	Curcumin	11.2	/	(1) Inhibits oxidative stress (2) Suppression of Apoptosis	DM mouse model (STZ)	A	[79]

PNs: Polymeric nanoparticles; EE: encapsulation efficiency; DLC: drug loading capacity; ADR: adriamycin; PHN: passive Heymann nephritis; IgAN: IgA nephropathy; STZ: streptozotocin; DM: diabetes mellitus; C: *in vitro* only; A: *in vivo* only; C + A₁: *in vitro* + single animal model; C + A_2_: *in vitro* + two animal models.

#### 3.1.3. INs: Nanozyme activity and physical effects

IN leverage intrinsic properties of metal oxides (e.g. CeO_2_-ZrO_2_, ZnO) or carbon-based materials (e.g. graphene quantum dots) for therapeutic action, optionally combined with drug loading for synergistic effects [70–73]. For instance, 7CZ nanoparticles (9 nm) mimic superoxide dismutase (SOD) activity was carried out *via* Ce³^+^/Ce^4+^ redox cycling, directly scavenging ROS and restoring podocyte cytoskeletal integrity [64]. IN offer advantages such as long-lasting antioxidant capacity and simplified fabrication (no drug-loading required), but long-term biosafety concerns (e.g. metal ion accumulation) must be addressed through degradable coatings or chelating agents (Table 3).

**Table 3.** INs nanomaterials with podocyte protection effects.

**Core composition**	**Size (nm)**	**Main targeting strategy**	**Drug loading**	**EE (%)**	**DLC (%)**	**Main mechanism of action**	**Verification model**	**Experimental phase**	**Refs.**
7CZ	9	Size and ROS targeting	/	/	/	(1) Stabilize cytoskeleton (2) Suppression of apoptosis	(1) Podocyte(ADR) (2) Mouse model (ADR)	C + A₁	[70]
AuNPs	51.8 ± 0.7	Size targeting	/	/	/	(1) Inhibition of inflammation (2) Inhibits oxidative stress	DM mouse model (STZ)	A	[71]
GQDs	2.5 ± 0.5	Size targeting	/	/	/	(1) Regulation of Ca²⁺ homeostasis (2) Antioxidant and anti-apoptotic effects (3) Restoration of mitochondrial function	(1) Podocyte (H_2_O_2_-induced) (2) ADN mouse model (3) 5/6Nx Rat model	C + A_2_	[72]
ZnONPs	< 100	Size targeting	/	/	/	(1) Reduction in GBM thickening (2) Increase in podocyte number (3) Restoration of nephrin and podocin expression	DM mouse model (STZ)	A	[73]

INs: Inorganic nanomaterials; EE: encapsulation efficiency; DLC: drug loading capacity; ADR: adriamycin; STZ: streptozotocin; 5/6Nx: 5/6 nephrectomy; ADN: adriamycin-induced nephropathy; C: *in vitro* only; A: *in vivo* only; C + A₁: *in vitro* + single animal model; C + A_2_: *in vitro* + two animal models.

### 3.2. Particle size, morphology, and surface charge of nanomedicines

The size, morphology, and surface charge of nanomedicines are critical parameters determining their bioactivity, stability, cellular uptake efficiency, and biodistribution [61,62]. In podocyte-protection studies, nanoparticles typically exhibit sizes around 100 nm (e.g. ZnO NPs, RGD-HSA-TAC NPs) [63,73], while smaller systems—such as spherical cerium-zirconium oxide nanomedicine (7CZ) with a diameter of ∼9 nm—demonstrate unique advantages. This nanoscale size and spherical morphology confer exceptional dispersion stability, preventing aggregation in physiological environments and ensuring stable circulation. Furthermore, optimal dimensions and morphology significantly enhance 7CZ uptake by podocytes, enabling efficient clearance of intracellular ROS and suppression of oxidative stress-mediated podocyte injury [70].

Surface charge is another pivotal factor influencing nanomedicine performance. Positively charged nanomaterials (e.g. PC-PLNs, zeta potential: +11.86 ± 0.95 mV) enhance binding to negatively charged glomerular cell surfaces *via* electrostatic interactions, improving cellular uptake [66]. However, positive charges may also increase nonspecific binding with blood components (e.g. negatively charged proteins), raising risks of immunogenicity and reduced hemocompatibility. In contrast, negatively charged systems exhibit superior serum stability [80]. For instance, cRGD-modified dendritic albumin nanocomplexes (zeta potential: −17.4 ± 1.08 mV) minimize nonspecific adsorption through their negative charge while achieving HDAC4 gene silencing *via* targeting ligands, effectively ameliorating podocyte injury in diabetic nephropathy models [64]. Additional advantages of negatively charged materials include reduced aggregation propensity and enhanced hemocompatibility. Nevertheless, their glomerular enrichment efficiency may lag behind positively charged systems, necessitating compensatory strategies such as targeting ligand engineering.

### 3.3. Encapsulation efficiency, drug loading capacity, and stability

EE, DLC, and stability are the critical parameters for evaluating nanomedicine performance. In the study of hyaluronic acid-functionalized Span-Labrasol nanovesicles (FRA-SP), the EE of ferulic acid (FRA) reached 61.2%–98.1%, with the optimized formulation FRA-L-H-SP achieving an EE of 98.1 ± 4.1%. This system demonstrated exceptional stability in *in vitro* release and *ex vivo* permeation experiments, enabling sustained FRA release to effectively protect kidney [81]. Similarly, the celastrol (CLT) delivery system (PC-PLNs) exhibited an EE of 94.83 ± 0.87% and a DLC of 2.83 ± 0.025%, achieving podocyte-targeted protection and therapeutic efficacy in chronic kidney disease (CKD) through high-efficiency drug loading [67].

Current nanomedicine systems generally exhibit DLC below 10%, with rare exceptions such as the cRGD-modified dendritic polymer nanocomplexes developed by Guo Z et al. which achieved a DLC exceeding 10% [63]. Low DLC remains a major challenge in the field, as existing high-loading systems predominantly rely on inert porous materials as drug carriers [82]. The development of novel high-DLC nanoparticles could reduce the quantity of carrier materials required, thereby minimizing the metabolic and excretory burden associated with excess non-therapeutic components.

### 3.4. Key limitations of renal-targeted nanodelivery systems in podocyte therapy

Some findings in current nanotherapy studies have provided important scientific evidence for the subsequent optimization of carriers and innovation in delivery strategies. First, insufficient intracellular drug release efficiency represents a core bottleneck restricting the therapeutic efficacy of nanodelivery systems. Taking BSA633-MP as an example, it enters podocytes *via* FcRn receptor-mediated endocytosis and releases drugs through acid-sensitive bond hydrolysis in the acidic environment of lysosomes, but the drug release rate is only 72% within 48 h. Such incomplete release results in a significantly weaker protective effect against PAN-induced podocyte apoptosis (20.07% increase in cell viability) compared with free methylprednisolone (41.36% increase). Nanocarriers undergo a multistep intracellular trafficking process including receptor recognition, endocytosis, and lysosomal transport, and the hydrolysis of chemical bonds hardly achieves complete drug release, ultimately leading to inferior therapeutic efficacy of the targeted system relative to free drugs [66].

Second, the size, structure, and DLC of nanocarriers exert significant dual effects on therapeutic outcomes, presenting a contradictory relationship of mutual restriction among multiple factors. There exists an optimal size window for glomerular filtration penetration and podocyte endocytosis: 60 nm chitosan/DNA nanocapsules exhibit significantly higher inhibition efficiency (84%) against c-mip gene in podocytes than 20-nm (52%) and 100-nm (30%) carriers. Particles that are too small suffer from insufficient ligand density for effective target binding, while excessively large ones fail to pass through the impaired glomerular filtration barrier [75]. A hydrophobic PCL core can improve the encapsulation efficiency of dexamethasone but simultaneously delays drug release, suggesting that a balance must be achieved between high drug loading and rapid release [76]. In the CS-PEG@CP1@1@Semaglutide system, the release rate is optimal when the drug loading is 20%. Further increasing the concentration fails to enhance the release rate due to saturation of drug-loading sites and strengthened intermolecular interactions.

Finally, the accumulation of nanocarriers in off-target organs poses potential long-term safety risks, representing a critical limiting factor for clinical translation. Significant hepatic enrichment of BSA633-MP is still observed at 24 h [66]. Ultrasmall polymeric nanocarriers show obvious accumulation in the spleen, which may interfere with the function of the reticuloendothelial system and immune cells. Although no acute toxicity is detected, their long-term biosafety still requires systematic evaluation [76].

Overall, the aforementioned limitations and contradictory findings regarding intracellular drug release, carrier properties, and off-target accumulation collectively highlight the critical challenges of renal-targeted nanodelivery systems, underscoring the necessity for further optimization of carrier design and administration strategies to promote their clinical translation.

## 4. Nanotechnology-mediated podocyte repair mechanisms

**Figure 2.**
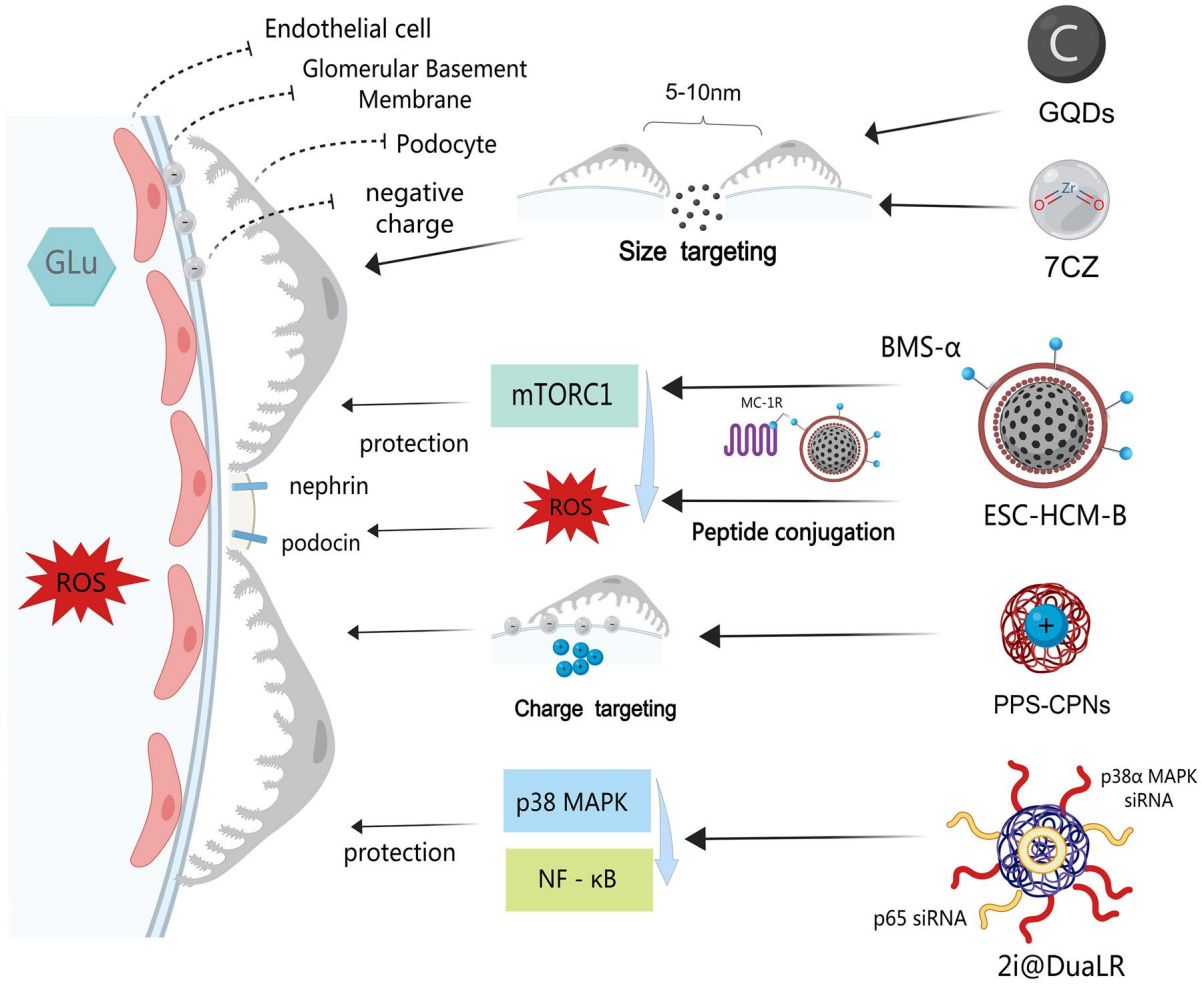
Schematic illustration of targeted protective mechanisms of nanomaterials on podocytes. This figure depicts three primary strategies employed by nanomaterials to target and protect glomerular podocytes: (1) Size-based targeting (passive targeting): ultrasmall nanoparticles, such as 9-nm 7CZ nanoparticles and 2.5 ± 0.5-nm graphene quantum dots (GQDs), can penetrate the GFB and reach the renal cortex and podocytes; (2) charge-based targeting (passive targeting): Positively charged nanoparticles (e.g. PPS-CPNs) can bind to the negatively charged glomerular basement membrane (GBM) *via* electrostatic attraction, leading to their accumulation in the glomerulus. (3) Active targeting: Nanoplatforms (e.g. ESC-HCM-B) are functionalized with specific ligands (e.g. BMS-α, a ligand for MC-1R) to recognize and bind to receptors on podocytes, facilitating receptor-mediated endocytosis.These targeted nanomaterials exert protective effects on podocytes by modulating key signaling pathways. For example, ESC-HCM-B activates the cAMP-PKA pathway to reduce inflammation, while 2i@DuaLR loaded with p65 and p38α MAPK siRNAs inhibits the NF-κB and p38 MAPK pathways, thereby mitigating apoptosis and inflammation.

Podocyte injury represents a central pathological event in the progression of various kidney diseases. Nanotechnology offers innovative solutions for podocyte protection through precision delivery systems (Figure 2).

### 4.1. Targeted delivery strategies

#### 4.1.1. Active targeting

Active targeting relies on the conjugation of ligands to nanoparticles to enable the recognition of specific biomarkers on podocytes. For instance, the ESC-HCM-B nanoplatform shown in Figure 2 achieves active targeting to glomerular podocytes by anchoring BMS-470539 (BMS-α), a specific ligand for the podocyte MC-1R, onto the nanoplatform surface. It further ameliorates podocyte inflammation by activating the cAMP-PKA signaling pathway [74]. In addition, the cRGD can specifically bind to the αvβ3 integrin receptor, which is highly expressed on the surface of injured podocytes, mediating receptor-dependent endocytosis of nanocomplexes and thus enabling targeted delivery to diseased podocytes. Depending on the loaded drug, different mechanisms are employed to exert the therapeutic effects. For example, upon loading with HDAC4 siRNA, gene silencing of HDAC4 can be achieved *via* RNA interference, inhibiting the deacetylation and nuclear translocation of STAT1, blocking apoptotic and inflammatory pathways, and alleviating podocyte injury [64,77].

#### 4.1.2. Passive targeting

Passive targeting mainly depends on the size selectivity of the GFB (endothelial fenestrae 70–90 nm) and the negative charge of the GBM. For example, the 9-nm 7CZ nanoparticles and 2.5 ± 0.5 nm GQDs shown in Figure 2 can rapidly penetrate the GFB and reach lesion sites such as the renal cortex and podocytes due to their ultrasmall size, and are readily endocytosed by cells to exert therapeutic effects [70,72]. Guo et al. designed three types of nanoparticles with varying positive charge intensities (CPN-10, CPN-20, and CPN-30), which can bind to the negatively charged GBM *via* electrostatic attraction and accumulate in this region. Among them, highly positively charged nanoparticles tend to form aggregates with plasma proteins and erythrocytes, which are then trapped in pulmonary capillaries and cleared by the reticuloendothelial system. In contrast, CPN-10 with the lowest positive charge (approximately 10 mV) contains more PPS-PEG, allowing the formation of a dense and stable PEG layer on its surface. This effectively prevents particle aggregation and enhances colloidal stability in serum, thereby prolonging blood circulation time, reducing accumulation in the liver and lungs, and extending renal retention [69].

Active targeting is superior to passive targeting in therapeutic specificity and efficacy for disease with clear molecular targets (e.g. DKD with high αvβ3 expression, IgAN with high VCAM-1 expression), and is the first choice for the design of PDCs and PNs with ligand modification; passive targeting is more suitable for podocyte injury with unclear molecular targets or extensive pathological changes (e.g. advanced glomerulosclerosis), and is the main targeting mode of INs relying on size/charge effects, with the advantage of covering a wider range of pathological scenarios.

### 4.2. Antioxidant and anti-inflammatory mechanisms

Nanotechnology plays a pivotal role in treating kidney diseases by blocking injury cascades through antioxidant and anti-inflammatory mechanisms.

#### 4.2.1. Nanozyme-mediated ROS scavenging

Nanozymes mimic natural antioxidant enzyme activities to scavenge ROS. For example, CeO_2_-ZrO_2_ nanoparticles exploit the Ce³^+^/Ce^4+^ redox cycle to exhibit dual enzymatic activities of SOD and catalase, efficiently eliminating excess ROS while restoring damaged mitochondrial membrane potential [70]. ZnO NPs enhance the expression of antioxidant enzymes (e.g. SOD, glutathione peroxidase (GPx)) *via* activation of the Nrf2/HO-1 pathway, concurrently suppressing the TXNIP/NLRP3 pyroptosis pathway to alleviate oxidative stress-induced podocyte injury [83]. In membranous nephropathy models, ROS-responsive PPS-CPNs/CLT nanosystems significantly ameliorate pathological conditions by depleting excessive ROS [69]. These nanomaterials provide diversified therapeutic strategies for ROS-related renal injury through synergistic multi-pathway interactions.

#### 4.2.2. Inflammatory signaling modulation

Nanomedicines enable precise regulation of inflammatory signaling pathways. Celastrol-loaded PC-PLNs attenuate inflammatory responses by downregulating TNF-α expression, blocking NF-κB nuclear translocation, and inhibiting IL-6 secretion [67]. Silibinin nanoliposomes employ a dual regulatory mechanism: downregulating the pro-fibrotic TGF-β1/Smad2/3 pathway while upregulating antagonistic Smad6/7 molecules, thereby maintaining TGF-β/Smad signaling balance and effectively inhibiting renal fibrosis progression [84]. These studies demonstrate that nanotechnology offers innovative strategies to halt kidney disease progression *via* multi-target interventions in inflammatory cascades.

Nanozyme-mediated ROS scavenging is a fundamental antioxidant mechanism for podocyte injury with excessive ROS accumulation, and is the main therapeutic mode of INs (e.g. 7CZ, GQDs, ZnONPs); inflammatory signaling modulation is a precision anti-inflammatory mechanism for podocyte injury dominated by specific inflammatory pathway activation, and is mostly combined with active/passive targeting by PDCs and PNs to achieve synergistic therapy. The combination of the two mechanisms has a superior therapeutic effect to single mechanism, which can block the mutual promotion of oxidative stress and inflammation, and is the optimal choice for the design of multifunctional nanomedicines.

Summary: Targeted delivery strategies (especially active targeting) are the core and most effective repair mechanisms for nanotechnology-mediated podocyte injury repair, which determine the precision of nanomedicine therapy; antioxidant and anti-inflammatory mechanisms are the universal and indispensable supplementary mechanisms, which expand the applicable scenarios of nanomedicine and enhance the therapeutic effect by blocking common pathological cascades. In practical nanomedicine design, the synergistic application of targeted delivery and antioxidant/anti-inflammatory mechanisms is the mainstream direction, and its therapeutic efficacy is significantly higher than that of a single mechanism; while a single passive targeting or antioxidant mechanism is only suitable for mild or specific podocyte injury, with the limited clinical application potential.

## 5. Current challenges and future directions

**Figure 3.**
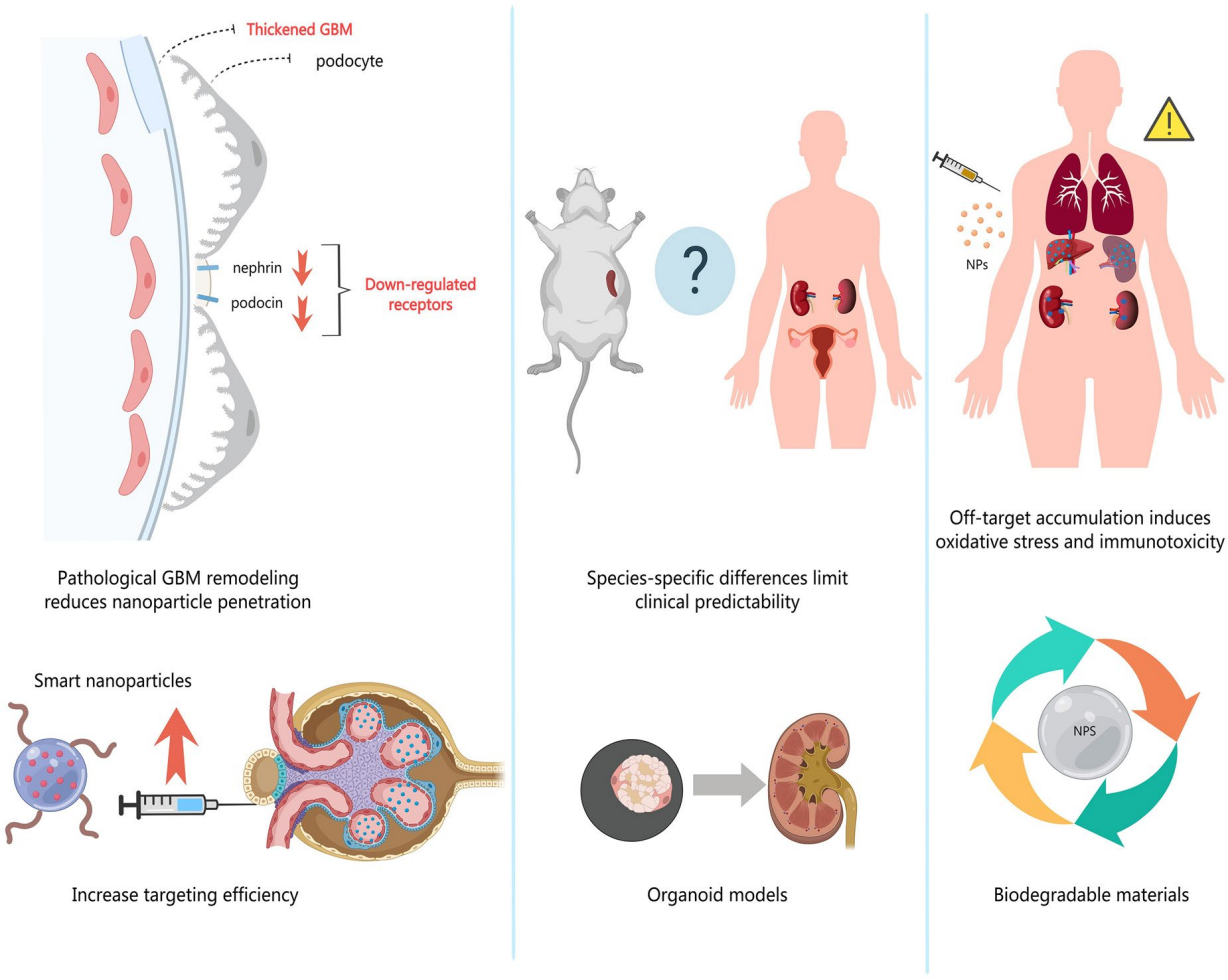
Conceptual schematic of the key clinical translation challenges and corresponding strategies for podocyte-targeted nanomaterials. This schematic illustrates three core translational barriers (optimization of targeting efficiency, species-specific differences between animal models and humans, and toxicity induced by off-target accumulation) and their corresponding optimization strategies (smart nanoparticles for enhanced targeting efficiency, organoid models for improved clinical predictability, and biodegradable materials for reduced biosafety risks).

Despite the significant therapeutic potential of nanotechnology in podocyte injury repair, the clinical translation and practical application in this field still face multidimensional challenges, which can be prioritized by their urgency of restricting clinical translation and mainly fall into three core categories: the most urgent core barriers that directly block the transition from preclinical research to clinical trials, secondary key challenges that affect the long-term efficacy and safety of clinical application, and supporting technical bottlenecks that restrict industrialization and large-scale clinical use. Specifically, the most urgent core barriers refer to the poor clinical predictability of preclinical animal models and the low targeting efficiency of nanomaterials caused by biological barrier remodeling; the secondary key challenges are the long-term biosafety risks of nanomaterials such as inorganic nanomaterial accumulation and immunogenicity; the supporting technical bottlenecks include the batch-to-batch inconsistency in scalable manufacturing and the lack of standardized quality control systems. These challenges are hierarchically interrelated, and breaking the most urgent core barriers is the primary prerequisite for advancing the clinical translation of podocyte-targeted nanomedicines **(**Figure 3**).**

### 5.1. Targeting efficiency and biological barriers

#### 5.1.1. Pathological remodeling of the GBM

Pathological remodeling of the GBM constitutes a major biological barrier for nanodrug targeting. In chronic kidney diseases such as DKD and GBM thickening—an early critical pathological feature—arises from dysregulated matrix modulation and AGE accumulation [85]. This structural alteration significantly reduces the permeation efficiency of conventional drugs and disrupts the size-dependent retention mechanism of nanoparticles. Furthermore, downregulation of podocyte-specific surface markers (e.g. nephrin, podocin) under disease conditions diminishes the active targeting efficiency of ligand-modified nanoparticles [86], further compromising therapeutic efficacy.

#### 5.1.2. off-target organ accumulation and detrimental effects

Nanoparticles in systemic circulation are prone to recognition and clearance by the mononuclear phagocyte system (MPS), leading to nonspecific accumulation in organs such as the liver and spleen [87,88]. Chronic accumulation may induce oxidative stress and mitochondrial dysfunction [89]. Surface modification strategies (e.g. zwitterionic coatings or erythrocyte membrane mimics) reduce serum protein adsorption, prolong circulation time, and mitigate off-target organ toxicity.

### 5.2. Long-term safety and biocompatibility

#### 5.2.1. Metabolism and toxicity of inorganic nanomaterials

Metal oxide nanoparticles (MONPs) have demonstrated significant potential in antioxidant and anti-inflammatory therapies due to their unique physicochemical properties, such as a high surface area-to-volume ratio and excellent ligand-binding capacity [90]. However, their widespread application is hindered by substantial biohazard risks and potential toxicity associated with long-term retention [91]. The toxicity of MONPs is primarily mediated through two core mechanisms: ROS-dependent pathways and non-ROS-dependent pathways [91].

Activation of ROS induces oxidative stress, leading to lipid peroxidation, cell membrane damage, protein denaturation, and DNA damage. Furthermore, it can trigger the apoptotic pathway by activating caspase-9 [91]. Non-ROS-mediated toxicity includes cytotoxicity caused by released metal ions, excessive accumulation of nanoparticles on the cell surface, and specific binding to death receptors [91]. For example, sustained extracellular zinc ion exposure may induce cytotoxic insults in cortical collecting duct (CCD) principal cells, characterized by increased cell death and disrupted cytoskeletal organization[92]. Similarly, high doses of TiO_2_ nanoparticles can accumulate in the liver, spleen, and lungs of organisms, resulting in organ edema, inflammatory cell infiltration, and impaired organ function, with young individuals being more susceptible [91].

To address these safety concerns, developing biodegradable organic–inorganic hybrid materials or introducing chelating ligands to promote metal ion excretion is a potential strategy [90,91]. Additionally, surface modification (e.g. coating with ethylenediaminetetramethylene phosphonic acid) can reduce oxidative stress-induced cytotoxicity, and optimizing the size, shape, and surface properties of nanoparticles may alleviate their adverse effects [90]. Furthermore, evaluating the combined toxicity in complex systems (such as interactions between ZnO and TiO_2_ nanoparticles or with other food additives) is crucial for accurate safety assessment. This is because the physicochemical properties of MONPs may undergo significant changes in mixed environments, thereby altering their toxicity [91].

#### 5.2.2. Immunogenicity and complement activation risks

Liposomal and polymeric nanocarriers may provoke immunotoxicity by activating the complement system or eliciting anti-carrier antibody responses. For example, liposomes can trigger complement cascade activation, exacerbating inflammatory responses [93,94]. Optimizing carrier design through surface charge modulation or biomimetic membrane modifications effectively reduces immunogenicity risks [95].

### 5.3. Translational medicine and clinical adaptability

At present, the research and development of nanomaterials for podocyte injury repair is generally in the stage of conceptual verification to preclinical validation, with notable variations in the extent of experimental verification among different materials, which can be specifically categorized into three classes. Class 1 refers to materials with only a single model verified and thus the lowest level of evidence. This class includes two subcategories: One consists of materials tested solely in *in vitro* cellular assays, such as BSA633-MP of peptide-drug conjugates (PDCs) and CS-PEG@CP1@1@Semaglutide of polymeric nanoparticles (PN), which have confirmed anti-inflammatory, anti-apoptotic, and other reparative effects in *in vitro* podocyte models yet lack *in vivo* experimental validation data [66,78]; the other subcategory comprises materials validated only in animal models, for example, nCUR of PN as well as AuNPs and ZnONPs of inorganic nanomaterials (IN), whose efficacy has been verified merely in streptozotocin (STZ)-induced diabetic nephropathy animal models without corresponding *in vitro* cellular experiments [65,67,73]. Class 2 represents materials validated by both *in vitro* cellular assays and a single animal model, which are the mainstream of current research with a moderate level of evidence. As the major research focus, these materials have all been proven effective through combined *in vitro* and *in vivo* experiments, with the animal models mostly being mice or rats with a single injury type such as STZ-induced diabetic nephropathy, adriamycin (ADR)-induced nephrotic syndrome, and passive Heymann nephritis. Such verification has preliminarily demonstrated the *in vivo* targeting ability and reparative efficacy of these nanomaterials. Class 3 involves materials validated by both *in vitro* cellular assays and multiple animal models, boasting the highest level of evidence. This category includes PC-PLNs and ESC-HCM-B of PDCs, 2i@DuaLR of PN and GQDs of IN, all of which have been validated through *in vitro* cellular experiments combined with two or more small animal models [67,68,72,74]. This comprehensive verification has fully confirmed the stability and universality of their reparative effects, laying a solid experimental foundation for subsequent advanced preclinical research. However, the clinical translation of these nanomaterials is still hindered by two key issues: the inconsistency between preclinical animal models and human pathological characteristics, and the batch-to-batch variation in large-scale nanomaterial manufacturing.

#### 5.3.1. Discrepancies between animal models and human disease pathogenesis

Current animal models, such as STZ-induced diabetic rodents, inadequately recapitulate the molecular mechanisms underlying human podocyte injury. The STZ model directly destroys pancreatic β-cells *via* chemical toxicity, leading to absolute insulin deficiency and rapid-onset hyperglycemia [96]. In contrast, podocyte injury in human DKD is driven by multifactorial mechanisms, including chronic hyperglycemia, insulin resistance, AGE accumulation, and persistent inflammation [97]. Advanced strategies such as genetically engineered humanized mouse models or patient-derived podocyte organoids may enable more precise evaluation of nanotherapies’ clinical potential.

#### 5.3.2. Quality control challenges in scalable manufacturing

The quality control of large-scale production of nanomaterials faces multidimensional and complex challenges, with the core contradiction focusing on the coordinated imbalance between batch production and precise quality control. On one hand, there are significant variations in physicochemical properties (such as size, zeta potential, and impurity content) among different batches, with prominent risks of metal impurity residues and endotoxin contamination. Traditional detection methods are susceptible to interference from particle agglomeration or matrices: dynamic light scattering (DLS) exhibits significant deviations in size measurement of agglomerated materials, while manual analysis using transmission electron microscopy (TEM) is complex and prone to large errors [98]. Meanwhile, the lack of cross-scale mechanistic models and standardized databases linking microscale synthesis mechanisms to macroscale production hinders intelligent regulation [99], seriously affecting quality consistency. On the other hand, traditional synthesis methods have inherent limitations such as poor batch stability and difficulty in scaling up. Emerging technologies like microfluidics face reduced mass and heat transfer efficiency during scaling, and challenges including channel clogging and equipment compatibility further restrict process stability. The absence of real-time online detection technology prevents timely feedback and process adjustments, making it difficult to avoid the risk of substandard quality in batch products [100]. In addition, issues such as toxic interference from residual manufacturing components, biocompatibility risks, and the disconnect between evaluations of *in vivo* stability and drug release rates from physiologically relevant conditions [101] further exacerbate the complexity of quality control. Targeted technological optimization and the establishment of standardized systems are urgently needed to achieve breakthroughs.

### 5.4. Future directions

The advancement of nanotechnology in podocyte injury therapeutics will focus on five strategic dimensions to foster the integration of precision medicine and translational applications:

#### 5.4.1. Intelligent delivery system upgrades

Developing stimuli-responsive nanocarriers capable of sensing pathological microenvironmental cues (e.g. oxidative stress, aberrant enzymatic activity, or pathological metabolite accumulation) to dynamically modulate their size, surface charge, and drug release kinetics. Such systems will overcome the penetration limitations of conventional delivery methods across biological barriers, enhancing spatiotemporal precision, targeting efficiency, and lesion accumulation.

#### 5.4.2. Multimodal synergistic therapy platforms

Constructing multifunctional integrated nanoplatforms that synergize targeted delivery, gene regulation, immunomodulation, and physical interventions (e.g. photothermal/sonodynamic therapies) through orthogonal therapeutic mechanisms. This approach addresses the limitations of single-target therapies, comprehensively improving podocyte protection and regenerative repair efficacy.

#### 5.4.3. Biomimetic innovations and model systems

Establishing customizable humanized kidney models *via* organoid cultures, organ-on-a-chip technologies, and AI-driven predictive systems to recapitulate the dynamic progression of podocyte injury. These systems will accelerate high-throughput screening and efficacy validation of nanomedicines, providing precise predictive tools for personalized therapeutic design.

#### 5.4.4. Sustainable technology transformation

Advancing green synthesis protocols and biodegradable materials using strategies such as biomimetic mineralization, photocatalytic synthesis, and waste resource utilization to reduce energy consumption and environmental impact in nanomedicine production. Concurrently, developing metabolic tracking technologies and long-term safety evaluation frameworks will facilitate the construction of an eco-closed-loop system for clinical translation.

#### 5.4.5. Establishment of kidney-specific nanotoxicology research system

Given that the kidney is the main *in vivo* clearance organ of nanomaterials, a specialized kidney-specific nanotoxicology research system must be established to fill the gap of conventional toxicological research lacking organ-specific targeting. This system will focus on the unique renal physiological microenvironment, clarifying the interaction mechanism between nanomaterials (especially inorganic nanomaterials) and renal tissues/cells during filtration, reabsorption, and excretion; investigating the toxicological effects and molecular mechanisms of nanomaterial accumulation in renal interstitium and renal tubules on renal function and tissue homeostasis; and formulating kidney-specific nanomaterial safety evaluation criteria and threshold values to provide a rigorous scientific basis for the clinical translation of kidney-targeted nanomedicines.

The synergistic advancement of these directions will rely on interdisciplinary convergence of materials science, synthetic biology, clinical medicine, toxicology and data science, ultimately enabling rapid translation from foundational research to clinical practice, and ushering in a new era of precision intervention for kidney diseases, while ensuring the long-term safety and biocompatibility of nanomedicines in renal applications.

## Data Availability

Data sharing is not applicable to this article as no new data were created or analyzed in this study.
